# Development and preliminary validation of a virtual reality memory test for assessing visuospatial memory

**DOI:** 10.3389/fnagi.2023.1236084

**Published:** 2023-11-23

**Authors:** Ko Woon Kim, Jong Doo Choi, Juhee Chin, Byung Hwa Lee, Jee Hyun Choi, Duk L. Na

**Affiliations:** ^1^Department of Neurology, Jeonbuk National University Medical School and Hospital, Jeonju, Republic of Korea; ^2^Research Institute of Clinical Medicine of Jeonbuk National University-Biomedical Research Institute of Jeonbuk National University Hospital, Jeonju, Republic of Korea; ^3^Technology Development, Seers Technology Company Ltd., Seongnam-si, Republic of Korea; ^4^Department of Neurology, Samsung Medical Center, Sungkyunkwan University School of Medicine, Seoul, Republic of Korea; ^5^Center for Neuroscience, Korea Institute of Science and Technology, Seoul, Republic of Korea; ^6^Cell and Gene Therapy Institute(CGTI), Research Institute for Future Medicine, Samsung Medical Center, Seoul, Republic of Korea; ^7^Alzheimer’s Disease Convergence Research Center, Samsung Medical Center, Seoul, Republic of Korea

**Keywords:** Alzheimer’s disease, virtual reality, spatial memory, spatial navigation, head mounted display

## Abstract

**Background:**

Visuospatial memory impairment is a common symptom of Alzheimer’s disease; however, conventional visuospatial memory tests are insufficient to fully reflect visuospatial memory impairment in daily life.

**Methods:**

To address patients’ difficulties in locating and recalling misplaced objects, we introduced a novel visuospatial memory test, the Hidden Objects Test (HOT), conducted in a virtual environment. We categorized HOT scores into prospective memory, item free-recall, place free-recall, item recognition, and place-item matching scores. To validate the VR memory test, we compared HOT scores among individuals with Alzheimer’s disease (AD), amnestic mild cognitive impairment (aMCI), and normal controls (NC), and also compared these scores with those of conventional neuropsychological tests. We tracked the participants’ movement paths in the virtual environment and assessed basic features, such as total distance, duration, and speed. Additionally, we performed walking trajectory pattern mining such as outlier and stay-point detection.

**Results:**

We designed and implemented the HOT to simulate a house’s living room and assess participants’ ability to locate hidden objects. Our preliminary results showed that the total HOT score differed among 17 patients with AD, 14 with aMCI, and 15 NC (*p* < 0.001). The total HOT score correlated positively with conventional memory test scores (*p* < 0.001). Walking trajectories showed that patients with AD and aMCI wandered rather than going straight to the hidden objects. In terms of basic features, the total duration was significantly greater in AD than in NC (*p* = 0.008). In terms of trajectory pattern mining, the number of outliers, which were over 95% of the estimated trajectory, was significantly higher in AD than in NC (*p* = 0.002). The number of stay points, an index in which participants stayed in the same position for more than 2 s, was significantly higher in patients with AD and aMCI compared with NC (AD vs. NC: *p* = 0.003, aMCI vs. NC: *p* = 0.019).

**Conclusion:**

The HOT simulating real life showed potential as an ecologically valid test for assessing visuospatial memory function in daily life. Walking trajectory analysis suggested that patients with AD and aMCI wandered rather than going straight toward the hidden objects.

## 1 Introduction

Patients with Alzheimer’s disease (AD) frequently complain that they lose or misplace objects and are unable to find them because of visuospatial memory impairment. However, impairment in visuospatial and visuospatial memory functions has not received as much focus as verbal memory function (Coughlan et al., 2018). There are several conventional visuospatial and visuospatial memory tests such as the Rey-Osterrieth Complex Figure Test (Cherrier et al., 1999) and the Benton Visual Retention Test (Benton, 1945), but they fail to capture many important aspects of visuospatial memory impairment in daily life. Therefore, ecologically valid visuospatial memory tests reflecting cognitive function in daily life are required.

Conventional pencil-and paper-based tests are designed to assess specific cognitive domains in a context that reflects an academic testing environment, whereas virtual reality (VR) has the potential to measure cognition with less potential bias by simulating real-world settings (Liu et al., 2019). Previous studies have developed ecologically valid VR scenarios including shopping tasks (Mrakic-Sposta et al., 2018; Porffy et al., 2022), social event tasks (Kim et al., 2018), driving tasks (Plancher et al., 2012), and a series of real-world tasks (Atkins et al., 2018). Given concerns about performance differences in an artificial environment, these ecologically valid contexts aimed to be patient-friendly, reducing discomfort, and enhancing motivation.

VR is an optimal tool to overcome the limitations of conventional pencil-and paper-based visuospatial memory assessments (Jonson et al., 2021). Previous studies have developed virtual navigation tasks to test spatial orientation ability using the VR maze (Weniger et al., 2011; Parizkova et al., 2018), road map (Morganti et al., 2013), and hidden goal task (Serino et al., 2015; Bazadona et al., 2020) which can be a human version of the Morris water maze task (Morris, 2015). The majority of VR tests focus on way finding. While that is a common complaint, difficulty finding previously placed common objects in the home environment presents unique challenges that have not been directly examined. Therefore, we developed a visuospatial memory test using VR, called the Hidden Objects Test (HOT), based on patients’ complaints of losing or misplacing objects and their inability to find them. This test was designed to memorize hidden objects and their locations and to find them in a virtual environment. Furthermore, digital behavioral data, such as movement distance, time, and rating scores were measured during the test.

To assess the validity of the HOT, we examined the HOT scores among individuals with AD, aMCI, and NC, as well as between amyloid-negative and amyloid-positive aMCI subgroups. Additionally, we investigated digital behavioral data during the VR test, such as tracking trajectory, to explore their potential as digital biomarkers.

## 2 Methods

### 2.1 Developing and implementing the VR memory assessment test

#### 2.1.1 Developing the VR memory assessment test

**Figure 1:**
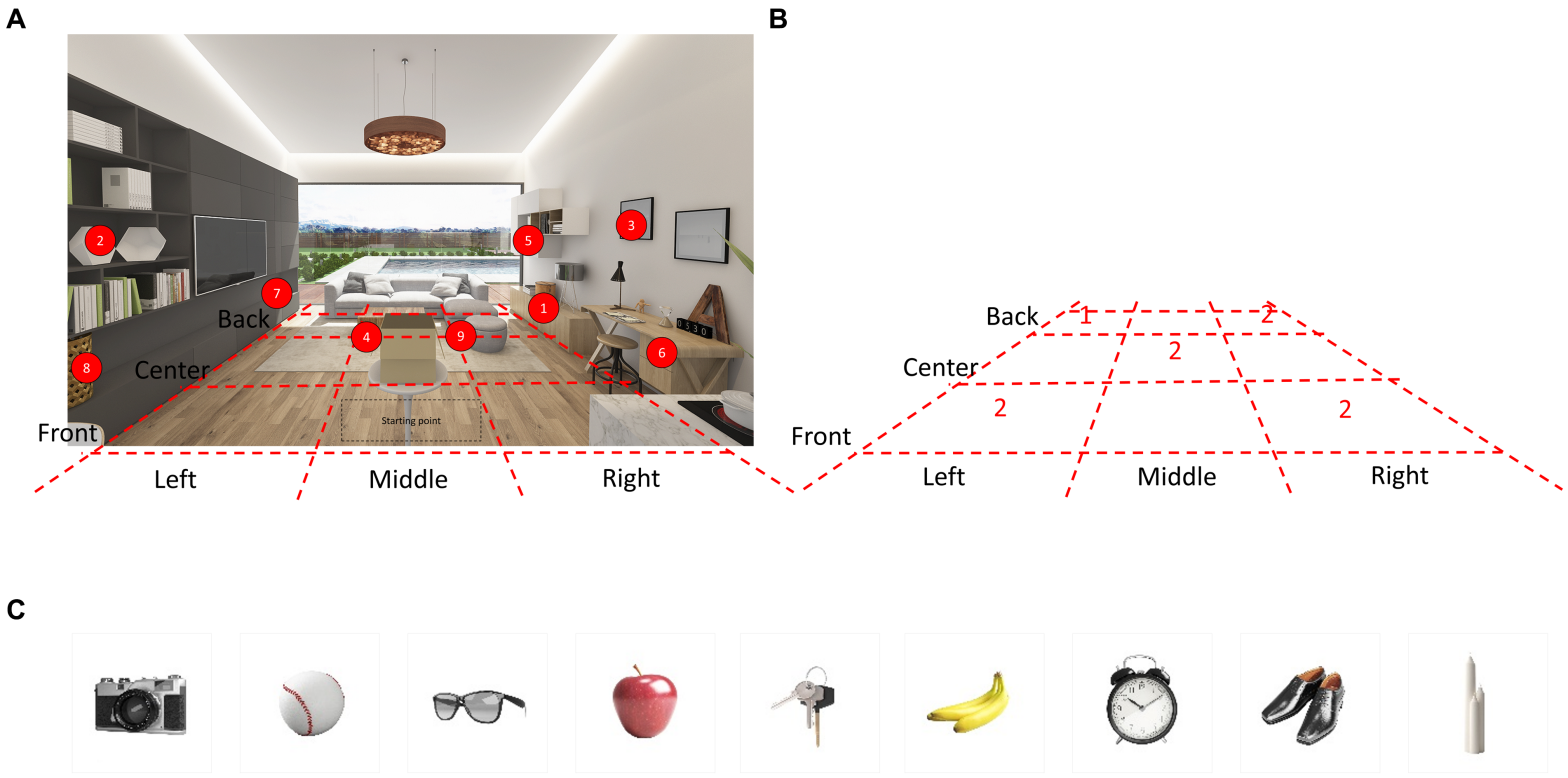
Virtual environment and hidden place. **(A)** The virtual environment consisted of a pleasant and comfortable living room with bright sunlight from the windows. A photorealistic virtual environment interface with 360-degree panoramas is rendered through the HMD. Nine items were hidden in different locations in the VR living room. Participants were allowed to walk freely around the space and open the furniture. **(B)** The VR living room was divided into nine sections based on the floor plan. Then, five spaces were selected out of the nine sections and configured each space to hide 1–2 items. **(C)** The images depict nine items hidden in various locations within the VR living room. HMD, Head Mounted Display; VR, Virtual Reality.

We developed a novel visuospatial memory test (Hidden Object Test; HOT; Korea Patent No. 10–1,983,279, 2019). The virtual environment consists of a pleasant and cozy living room with bright sunlight from a window (Figure 1A). A photorealistic virtual environment interface with 360-degree panoramas was created using the HMD. We divided the virtual living room into nine sections based on the floor plan, as shown in the Figure 1A. Then, we selected five spaces out of the nine sections and configured each space to hide 1–2 items, as shown in the Figure 1B. As a result, the hidden locations were evenly distributed within the virtual environment.

At the beginning of the test administration, participants are told three directions that they must execute at the end of the test as a prospective memory challenge. Subsequently, common household objects are presented in this virtual environment and placed in various locations for later recall. The final version of the HOT is comprised of the following five subtests (total possible score): *prospective memory test* (3), *item free-recall test* (9), *place free-recall test* (9), *item recognition test* (9), *and place-item matching test* (9). The total score for the five subtests is 39.

Common household objects were categorized into four groups: (1) tools and electronics, (2) foods, (3) wearable items, and (4) other items commonly found in households. We extracted the top 223 words from frequently used Korean vocabulary and categorized them accordingly (Supplementary Table S1). Within these categories, our research team carefully selected household objects of suitable size, ensuring that there were not too many items with similar semantic, orthographic, or visual characteristics, either within or between categories. A total of nine items were chosen for each subtest, with two or three items selected from each category. The final list of items is presented in Table 1 and Figure 1C.

**Table 1:** The list of items and their categories.

No.	Category	Item
1	Tools and electronics	Camera
2	Other things	Baseball
3	Wearable things	Glasses
4	Foods	Apple
5	Other things	Keys
6	Foods	Banana
7	Tools and electronics	Table clock
8	Wearable things	Shoes
9	Other things	Candle

In consideration of the number of items, our primary objective was to select a sufficient number of items that would pose a challenge for participants with normal cognitive function, making it unlikely for them to answer all items correctly. Simultaneously, we aimed to minimize testing time while effectively distinguishing between the three groups. To achieve this, we conducted pretests with AD and aMCI patients, as well as individuals without cognitive impairments (AD = 2; MCI = 9; NC = 8) to assess feasibility and validate the test design. We chose to use 3 directions for the prospective memory test and included 9 items for each of the other four subtests. Subsequently, we conducted a development test with a new group of participants (AD = 7; MCI = 6; NC = 10), none of whom had previously participated in the pretests. Detailed clinical information and statistical results of HOT scores for the development group are presented in Supplementary Table S2.

#### 2.1.2 Experimental apparatus

The experimental device used was the HTC Vive VR headset (HTC Corporation, Taoyuan City, Taiwan). Vive consists of a head-mounted display (HMD) headset (1,080 × 1,200 pixel display per eye, a field of view of approximately 110 °), two controllers held by each hand, and two infrared laser emitter units, called lighthouses. The Vive lighthouse is a laser-based tracking system. It tracks the user’s head and handheld controllers by generating reference points for the photosensor-attached headset and controllers to locate their positions while the user is free to walk around and to open and close furniture doors using the controllers.

We developed a VR test with a frame rate of at least 90 frames/s and a latency of less than 10 ms to ensure that elderly participants did not experience dizziness. Additionally, we implemented head movement tracking to synchronize the VR test with the participants’ actual movements, while also avoiding the scenario where only the VR environment moves, which could potentially induce discomfort.

#### 2.1.3 Implementing the VR memory assessment test

During the practice trial, the VR living room was shown, and the participants were allowed to freely walk around the space and open the furniture to become familiar with the environment. At the beginning of the HOT, an instruction panel for the *prospective memory test* appeared in front of the participant and following sentences were written on the panel: *“You will perform tests to find hidden objects. At the end of the test, when the instruction says that tests are all over, first, turn off the stove. Second, turn on the television. Third, take the present in the box.”* Then, another instruction panel and a box appeared in front of the participant and the following sentences were written on the panel: *“Now, a test to find hidden objects will be started. Please try to remember the name of objects that come out of the box and the place where the objects are hidden.”* In a virtual environment, an object was presented from the box and its name was announced loudly. The object then rotated 360 degrees for 5 s before being randomly hidden in one of the various drawers or cabinets scattered around the virtual room. The same process was repeated for nine objects one after the other. There were nine closed spaces for hiding objects (see numbers in Figure 1), and their doors or covers were flung open when the objects approached the hidden space, or when the participants’ hand controller was close to the hidden space. All participants performed the tests in the same order.

**Figure 2:**
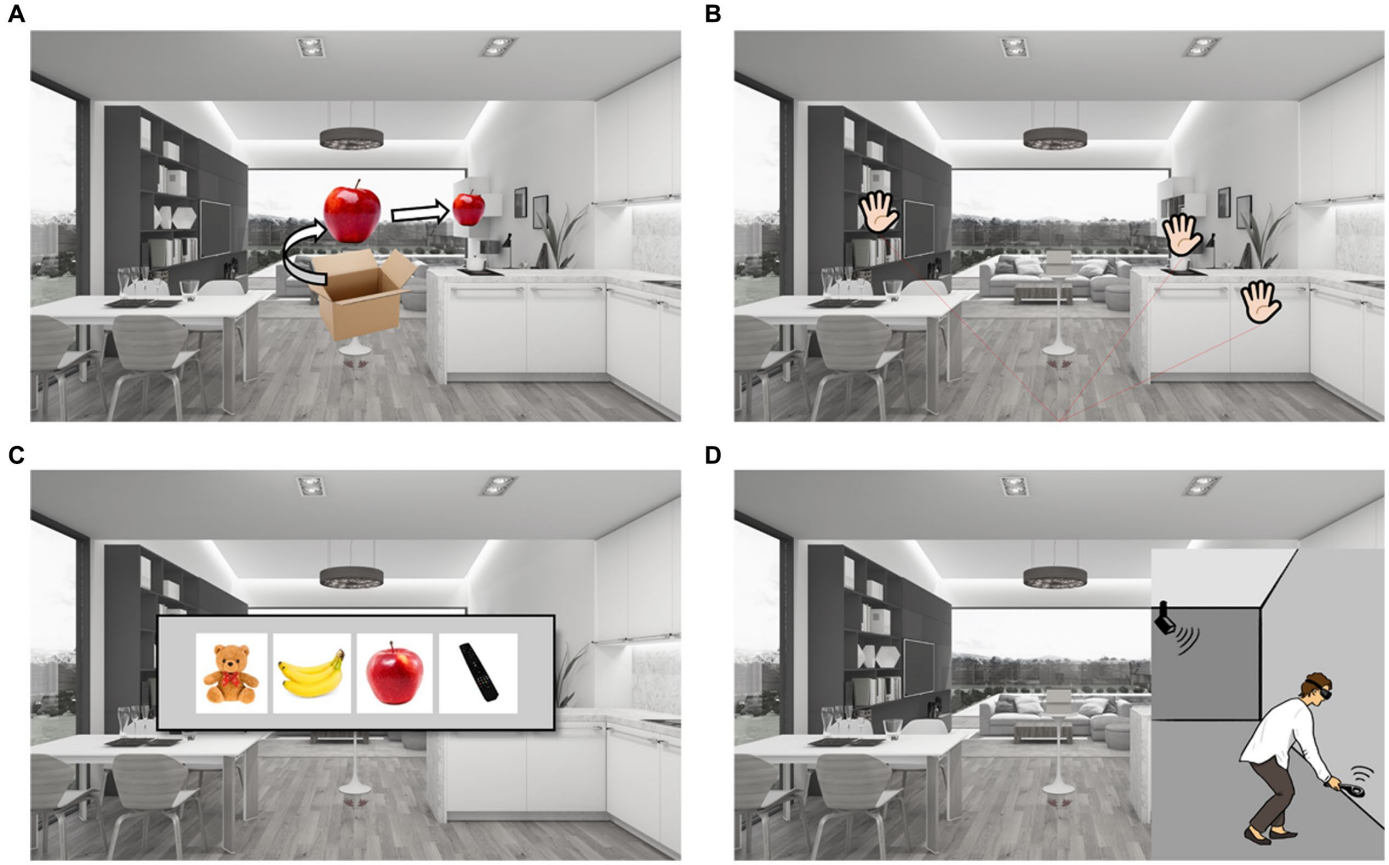
Experimental protocol. **(A)** Item free-recall test: items came out of the box and then turned 360 degree one by one, than the item moved into the each furniture space to be hided in order. Participants were instructed to say the names of nine items in any order. **(B)** Place free-recall test: participants were instructed to put palm-shaped stickers on the hidden place in any order. **(C)** Item recognition test: participants were instructed to select the hidden item among four items. **(D)** Place-item matching test: participants were instructed to walk into the virtual living room, and to reach the target furniture to find out hidden objects. Prospective memory test: participants were instructed to turn off the stove, to turn on the television, and to take the present in the box when the instruction says that all tests are over.

After completing the process of hiding nine items, participants were probed for memory under five different conditions, generating five subtest scores. In the *item free-recall test* (Figure 2A), participants were instructed to say the names of the nine items in any order. In the *place free-recall test* (Figure 2B), the participants were instructed to place palm-shaped stickers in a hidden place in any order. When placing the sticker, the participant did not approach the furniture but pointed at it with the controller like a laser-pointer and then pushed the button. In the *item recognition test* (Figure 2C), the participants were instructed to choose the correct answer from four given options. Three of the four options were not hidden. In the *place-item matching test* (Figure 2D), participants were instructed to walk into the virtual living room, reach the correct place, and find a hidden object when each item came out of the box. When the participants reached the correct place and grabbed the item using a controller, they were instructed to walk toward the box, the starting point, and then place the item into the box. The same process was repeated for all the nine items. After completion of all tests, the instruction sentence *“Tests are all over”* was displayed on the panel. Prospective memory performance was then measured based on the number of initial instructions executed.

### 2.2 Evaluating the validity of the VR memory test

#### 2.2.1 Recruitment of participants

Two groups of participants were recruited separately: a development group and a validation group. Both groups were consecutively selected from individuals who had visited the Memory Disorder Clinic at Samsung Medical Center in Seoul, Korea, between November 1, 2019, and January 30, 2020. A total of 23 participants were recruited in the development group, and subsequently, a total of 46 participants were recruited in the validation group. Participants in both groups were required to meet the following criteria: (i) Normal visual acuity, (ii) More than 1 year of education, (iii) Ability to walk unassisted, without the use of a gait aid, (iv) Completion of a standardized neuropsychological battery known as the Seoul Neuropsychological Screening Battery (SNSB) and the Mini-Mental State Examination (MMSE) test, and (v) Underwent magnetic resonance imaging (MRI). Note that the education criteria were waived in the development group.

In this study, we excluded participants with other structural lesions such as territorial infarction, intracranial hemorrhage, brain tumor, hydrocephalus, or severe white matter hyperintensities (WMH) observed on brain MR images. Severe WMH on MRI was defined as a cap or a band ≥10 mm and a deep white matter lesion ≥25 mm as modified from the Fazekas ischemia criteria. Experienced neurologists assessed the participants based on their clinical symptoms, medical/medication history reviews, neuropsychological test results, neuroimaging data, and laboratory tests.

Subsequently, the participants in both groups were categorized into AD, aMCI, and NC groups. All patients with AD met the probable AD dementia criteria proposed by the National Institute on Aging and the Alzheimer’s Association (NIA-AA) (Mckhann et al., 2011). We selected early-stage AD patients with mild dementia as defined by a clinical dementia rating (CDR) of 1. Note that one moderate-stage AD patient was included in the development group. All Patients with aMCI met the criteria proposed by Peterson and colleagues (Petersen, 2004). Subsequently, the aMCI group was divided into amyloid-negative [aMCI(−)] and amyloid-positive [aMCI(+)] groups based on amyloid PET positivity. NC participants were individuals who did not exhibit impairment on neuropsychological tests. The study was performed in accordance with the Declaration of Helsinki and was approved by the institutional review board of the Samsung Medical Center. Written informed consent was obtained from all the participants.

#### 2.2.2 Neuropsychological assessments

All participants underwent a standardized neuropsychological battery called the SNSB (Kang et al., 2012, 2019), which consists of tests for attention, language, calculation, visuospatial functioning, memory, and frontal-executive function, along with the MMSE and Clinical Dementia Rating (CDR) tests for general cognition. Each score was converted into a standardized Z-score based on age-and education-adjusted norms. Attention was assessed with the backward digit span and letter cancellation tests; language was tested using the Korean version of the Boston naming test (K-BNT); calculation was tested with three items each for addition, subtraction, multiplication, and division; visuospatial function was assessed with the Rey-Osterrieth Complex Figure Test (RCFT); memory function was assessed using immediate and delayed recall of the Seoul Verbal Learning Test (SVLT) and RCFT; and frontal-executive function was tested with the phonemic and semantic Controlled Oral Word Association Test (COWAT) and the Stroop word/color reading test.

#### 2.2.3 Amyloid PET scan

Amyloid PET scans were obtained using a Discovery STe PET/CT scanner (GE Medical Systems, Milwaukee, WI, United States) at the Samsung Medical Center using either a florbetaben (18F) (*n* = 8) or flutemetamol (18F) (*n* = 20) ligand. Amyloid PET positivity was interpreted based on the previously reported guidelines for each ligand (Barthel et al., 2011; Curtis et al., 2015).

#### 2.2.4 Measurement of walking trajectory

Walking trajectory in the virtual environment was tracked at a 13-Hz sampling rate. The virtual environment area was 3×4 m^2^. The participants started at the center of the virtual environment (coordinates: x = 12.01, y = 261.15, z = 0.00). The participants’ movement paths were assessed using total distance, duration, and mean speed. In addition to these basic walking metrics, we performed walking trajectory pattern mining to quantify the wandering patterns while searching for hidden objects.

#### 2.2.5 Measurement of walking trajectory pattern mining

To detect outliers in the walking trajectory, we used Kalman filtering, which is similar to the hidden Markov model (Kalman, 1960; Eddy, 1998). Kalman filtering uses sequential observations to calculate the posterior distribution derived by Bayesian inference. The walking trajectory was constructed based on the posterior distribution in the Bayesian implementation and walking observations with over 95% credible interval from the posterior distribution were annotated as *outliers*. We then measured *the distance of outliers* if we observed them sequentially.

We also identified a *stay point* where people remained in the same position using a *stay point detection* algorithm (Li et al., 2008). *Stay point detection* is an algorithm proposed to determine the location where a user stayed for a certain duration within a certain distance. *Stay point detection* algorithm requires distance and time threshold parameters. These threshold parameters were fixed at 2 s for the time threshold and 0.3 meters for the distance parameter. Therefore, a single stay point indicates that the participant remained stationary for 2 s within a distance of 0.3 meters.

#### 2.2.6 Statistical analysis

To assess demographics, cognitive profiles, behavioral data, and features of movement paths among the three groups, we used one-way ANOVA with pairwise multiple comparisons for continuous variables that satisfied the normal distribution and equality of variances. We used the Kruskal-Wallis test and the Mann–Whitney U test for continuous variables that did not satisfy the normal distribution and equality of variances. We performed *post hoc* comparisons using Dunn’s pairwise tests. We used the Kolmogorov–Smirnov test to assess the normal distribution of the variables. Levene’s test was used to assess the equality of variance of the variables. We used Pearson’s chi-square test for categorical variables, followed by Bonferroni correction. Pearson’s correlation was used to test the association between HOT scores and conventional neuropsychological test scores. The statistical software package SPSS version 23 was used for the data analyses. For all tests, the level of statistical significance was set at *p* < 0.05.

We performed one-way ANOVA with pairwise multiple comparisons to determine whether the number of outliers and stay points were significantly different across the disease groups. We used false discovery rate (FDR) correction for multiple comparisons in a principled manner.

## 3 Results

### 3.1 Demographics and neuropsychological assessments

The development group consisted of 7 patients with AD, 6 with aMCI, and 10 NC participants. Mean ± SD (min, max) ages were 72 ± 8 (63, 84) in the AD group, 78 ± 6 (68, 83) in the aMCI group, and 74 ± 7 (58, 83) in the NC group. Age was not significantly different between the three groups. Mean ± SD (min, max) CDR values were 1.1 ± 0.4 (1.0, 2.0) in the AD group, 0.5 ± 0.0 (0.5, 0.5) in the aMCI group, and 0.0 ± 0.0 (0.0, 0.0) in the NC group. Mean ± SD (min, max) MMSE scores were 19 ± 6 (6, 25) in the AD group, 26 ± 2 (24, 29) in the aMCI group, and 28 ± 2 (23, 30) in the NC group. CDR value (*p* < 0.001) and MMSE score (*p* = 0.001) were significantly different between the three groups. The demographic and cognitive profiles of the development group is summarized in Supplementary Table S2.

The validation group consisted of 17 patients with AD, 14 with aMCI, and 15 NC participants. Mean ± SD (min, max) ages were 69 ± 8 (52, 81) in the AD group, 71 ± 7 (64, 82) in the aMCI group, and 68 ± 8 (54, 82) in the NC group. Age was not significantly different between the three groups. Mean ± SD (min, max) CDR values were 1.0 ± 0.0 (1.0, 1.0) in the AD group, 0.5 ± 0.0 (0.5, 0.5) in the aMCI group, and 0.0 ± 0.0 (0.0, 0.0) in the NC group. Mean ± SD (min, max) MMSE scores were 21 ± 3 (17, 26) in the AD group, 25 ± 2 (21, 28) in the aMCI group, and 29 ± 1 (26, 30) in the NC group. Of the 46 validation group participants, 28 underwent amyloid positron emission tomography (PET) using either 18F-florbetaben PET (8/28) or 18F-flutemetamol PET (20/28) ligands, resulting in 10/13 patients with AD, 7/9 with aMCI, and 1/6 NC testing positive. CDR value (*p* < 0.001) and MMSE score (*p* < 0.001) were significantly different between the three groups. The demographic and cognitive profiles of the validation group is summarized in Table 2.

**Table 2:** Clinical information and cognitive profiles.

	Mean ± SD			*p* value *(Post hoc)*		
	AD (*N* = 17)	aMCI (*N* = 14)	NC (*N* = 15)	AD vs. aMCI	AD vs. NC	aMCI vs. NC
Age	69 ± 8	71 ± 7	68 ± 8	0.999	0.999	0.999
Sex (F:M)	9:8	10:4	11:4	0.852	0.819	0.999
Education	10.7 ± 5.1	11.3 ± 2.9	10.3 ± 6.0	0.999	0.999	0.999
APOE4 carrier^a^ %	44 (7/16)	64 (9/14)	29 (4/14)	0.261	0.397	0.063
Amyloid PET positivity %	77 (10/13)	78 (7/9)	17 (1/6)	0.586	*0.018*	0.060
MMSE	21 ± 3	25 ± 2	29 ± 1	*0.043*	*<0.001*	*0.006*
CDR	1.0 ± 0.0	0.5 ± 0.0	0.0 ± 0.0	*<0.001*	*<0.001*	0.999
CDR-SB	4.9 ± 1.8	1.6 ± 1.0	0.0 ± 0.0	*0.002*	*<0.001*	0.186
*Attention*						
Forward digit span z-score	−0.17 ± 1.12	0.29 ± 0.91	−0.17 ± 0.89	0.649	0.999	0.630
Backward digit span z-score	−0.40 ± 1.06	0.43 ± 1.48	−0.01 ± 1.36	0.352	0.999	0.999
*Language*						
K-BNT z-score	−1.33 ± 1.26	−0.14 ± 1.15	0.46 ± 0.73	*0.012*	*<0.001*	0.403
*Visuospatial function*						
RCFT: copying z-score	−0.36 ± 1.31	−0.40 ± 1.41	0.25 ± 0.3	0.999	0.424	0.394
*Memory*						
SVLT: delayed recall z-score	−2.57 ± 0.61	−1.72 ± 1.19	0.77 ± 0.68	*0.026*	*<0.001*	*0.001*
RCFT: delayed recall z-score	−2.36 ± 0.93	−1.04 ± 1.07	0.57 ± 0.87	*0.042*	*<0.001*	*0.015*
*Frontal/executive function*						
COWAT animal z-score	−1.15 ± 0.81	−0.33 ± 1.33	0.69 ± 0.98	0.222	*<0.001*	0.102
COWAT phonemic z-score	−0.29 ± 1.25	0.52 ± 1.70	1.23 ± 1.64	0.476	*0.023*	0.639
Stroop test: color z-score	−1.72 ± 2.01	−0.84 ± 1.11	0.51 ± 0.86	0.999	*<0.001*	*0.013*

ANOVA: age, digit-F, K-BNT, COWAT phonemic. Kruskal-Wallis test: education, MMSE, CDR, CDR-SB, digit B, RCFT copy, SVLT d, RCFT d COWAT animal, Stroop color. AD, Alzheimer’s disease; aMCI, amnestic mild cognitive impairment; APOE4, Apolipoprotein E4; CDR, Clinical Dementia Rating; CDR-SB, Clinical Dementia Rating Sum of Boxes; COWAT, Controlled Oral Word Association Test; K-BNT, Korean version of the Boston Naming Test; MMSE, Mini-Mental State Examination; NC, normal control; RCFT, Rey–Osterrieth Complex figure Test; SVLT, Seoul Verbal Learning Test. The italic values show significant differences at *p* values < 0.05.

a APOE4 expression was analyzed in 44 patients. Participants with one or more copies of the ε4 allele (i.e., ε2/4, ε3/4, and ε4/4) were considered ε4 carriers.

### 3.2 Comparison of HOT scores between groups

**Figure 3:**
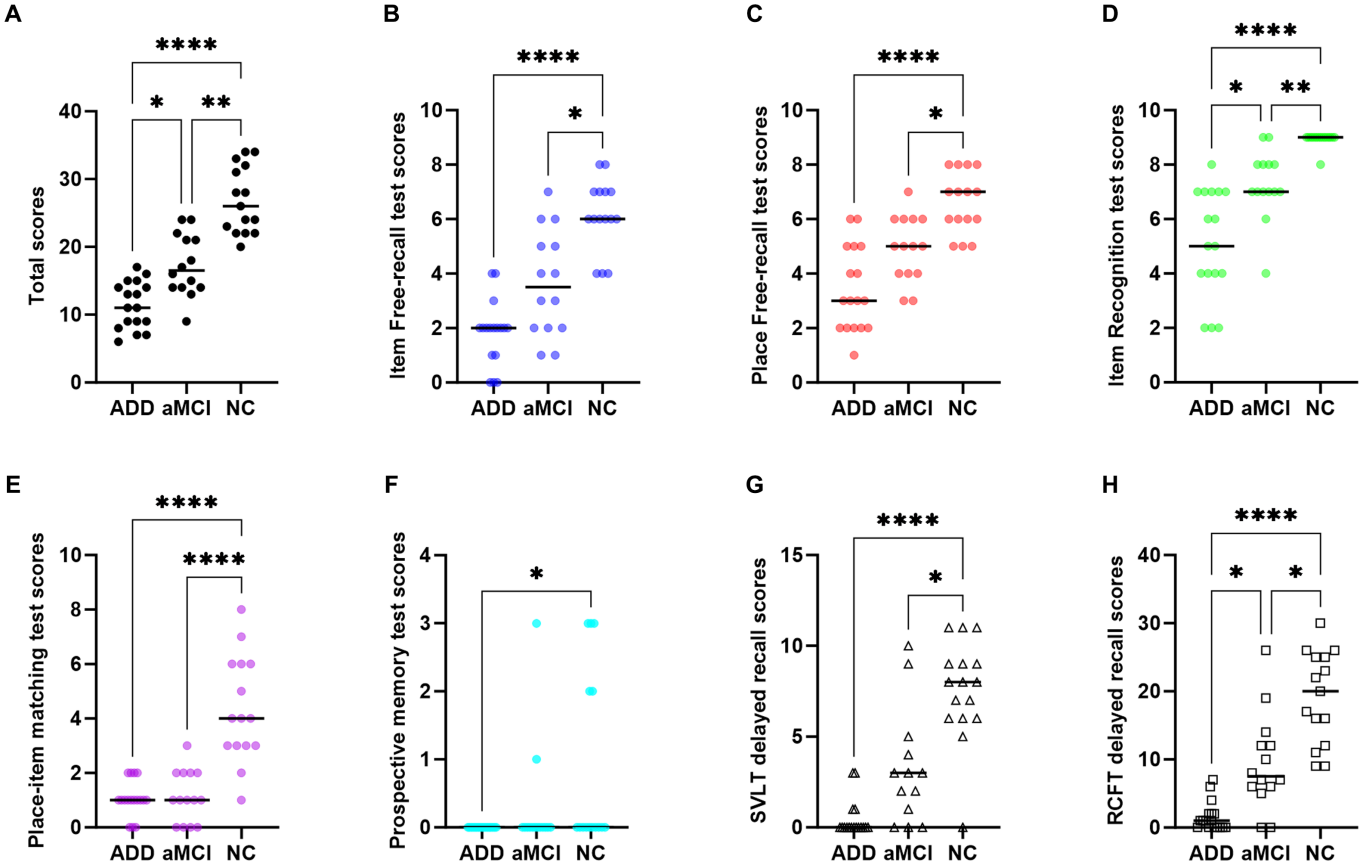
Scatter plots of HOT total scores and subtest scores. **(A)** Total scores, **(B)** Item-free recall test scores, **(C)** Place-free recall test scores, **(D)** Item recognition test scores, **(E)** Place-item matching test scores, and **(F)** Prospective memory test scores. **(G)** SVLT delayed recall scores, **(H)** RCFT delayed recall scores. Significance levels are indicated as follows: ^*^p < 0.05, ^**^p < 0.01, ^***^p < 0.001, ^****^p < 0.0001. RCFT, Rey–Osterrieth Complex figure Test; SVLT, Seoul Verbal Learning Test.

In the validation group, the mean total HOT score ± SD (min, max) was 11 ± 3 (6, 17) for patients with AD, 17 ± 5 (9, 24) for patients with aMCI, and 27 ± 5 (20, 34) for NC participants (Figure 3A); the difference was significant (*p* < 0.001); patients with AD performed worse than those with aMCI (*p* = 0.048), and those with aMCI performed worse than NC participants (*p* = 0.008) (Table 3). The five subtest scores differed significantly between the groups (*p* < 0.001). *Post hoc* test showed statistical significance as follows: Patients with AD and aMCI showed significantly lower scores than NC participants in the *item free-recall test* (AD vs. NC: *p* < 0.001, aMCI vs. NC: *p* = 0.015), the *place free-recall test* (AD vs. NC: *p* < 0.001, aMCI vs. NC: *p* = 0.032), the *item recognition test* (AD vs. NC: *p* < 0.001, aMCI vs. NC: *p* = 0.006), and the *place-item matching test* (AD vs. NC: *p* < 0.001, aMCI vs. NC: *p* < 0.001) (Figures 3B–F and Table 3). Patients with AD showed significantly lower scores than those with aMCI in the *item recognition test* (*p* = 0.049). Patients with AD showed significantly lower scores than NC participants in the *prospective memory test* (*p* = 0.027) (Figure 3F and Table 3). The total and five subtest scores were not significantly different between the amyloid-negative and amyloid-positive aMCI groups (Supplementary Table S3).

**Table 3:** Comparison of HOT scores among the three groups.

HOT subtests (total score)	Mean ± SD			*p* value *(Post hoc)*			AD (*N* = 17)	aMCI (*N* = 14)	NC (*N* = 15)	AD vs. aMCI	AD vs. NC	aMCI vs. NC
Item Free-recall test (9)	2 ± 1	4 ± 2	6 ± 1	0.089	*<0.001*	*0.015*
Place Free-recall test (9)	3 ± 2	5 ± 1	7 ± 1	0.103	*<0.001*	*0.032*
Item Recognition test (9)	5 ± 2	7 ± 2	9 ± 0	*0.049*	*<0.001*	*0.006*
Place-item matching (9)	1 ± 1	1 ± 1	4 ± 2	0.999	*<0.001*	*<0.001*
Prospective memory test (3)	0 ± 0	0 ± 1	1 ± 1	0.880	*0.027*	0.422
Total Score (39)	11 ± 3	17 ± 5	27 ± 5	*0.048*	*<0.001*	*0.008*

ANOVA: Item Free-recall. test.Kruskal-Wallis test: others. AD, Alzheimer’s disease; aMCI, amnestic mild cognitive impairment; HOT, Hidden Objects Test; NC, normal control. The italic values show significant differences at *p* values < 0.05.

### 3.3 Correlation analysis of HOT and neuropsychological test score

In the validation group, we compared the HOT and conventional neuropsychological test scores of patients with AD and aMCI (Table 4). The total HOT scores correlated positively with delayed recall in the SVLT (*r* = 0.631; *p* < 0.001), RCFT (*r* = 0.654; *p* < 0.001), Stroop word/color reading test (*r* = 0.392; *p* = 0.029), and MMSE (*r* = 0.511; *p* = 0.003) scores. Figures 3G,H shows the scatterplots of the SVLT and RCFT delayed recall scores to compare them with total and five subtest scores of HOT. The total HOT scores and five subtest scores did not show a significant correlation with age or education. The correlation *p* values between the five subtest scores and conventional neuropsychological test scores are shown in Table 4.

**Table 4:** The correlation coefficient between HOT and Neuropsychological test score in patients with AD and aMCI.

	Item free-recall test	Place free-recall test	Item recognition test	Place-item matching test	Prospective memory test	Total Score
Forward digit span	0.228	0.033	0.15	0.065	0.268	0.196
Backward digit span	0.196	0.369	0.243	−0.142	0.293	0.299
K-BNT	0.244	0.106	0.309	0.234	0.197	0.308
RCFT: copying	−0.023	0.309	0.315	0.322	0.157	0.288
SVLT: delayed recall	0.598^***^	0.397^*^	0.498^**^	0.381^*^	0.198	0.631^***^
RCFT: delayed recall	0.683^***^	0.400^*^	0.426^*^	0.322	0.457^*^	0.654^***^
COWAT animal	0.284	0.426^*^	0.228	−0.261	0.298	0.325
COWAT phonemic	0.188	0.267	0.256	−0.056	0.387^*^	0.293
Stroop test: color	0.381^*^	0.271	0.295	0.057	0.323	0.392^*^
MMSE	0.545^**^	0.314	0.380^*^	0.071	0.401^*^	0.511^**^
Age	−0.189	−0.217	−0.098	−0.169	−0.182	−0.200
Education	−0.068	0.057	0.149	0.154	0.058	0.080

^*^: *p* < 0.05, ^**^: *p* < 0.01, ^***^: *p* < 0.001. AD, Alzheimer’s disease; aMCI, amnestic mild cognitive impairment; HOT, Hidden Objects Test; NC, normal control. The italic values show significant differences at *p* values < 0.05.

### 3.4 Walking trajectory basic features and trajectory pattern mining

**Figure 4:**
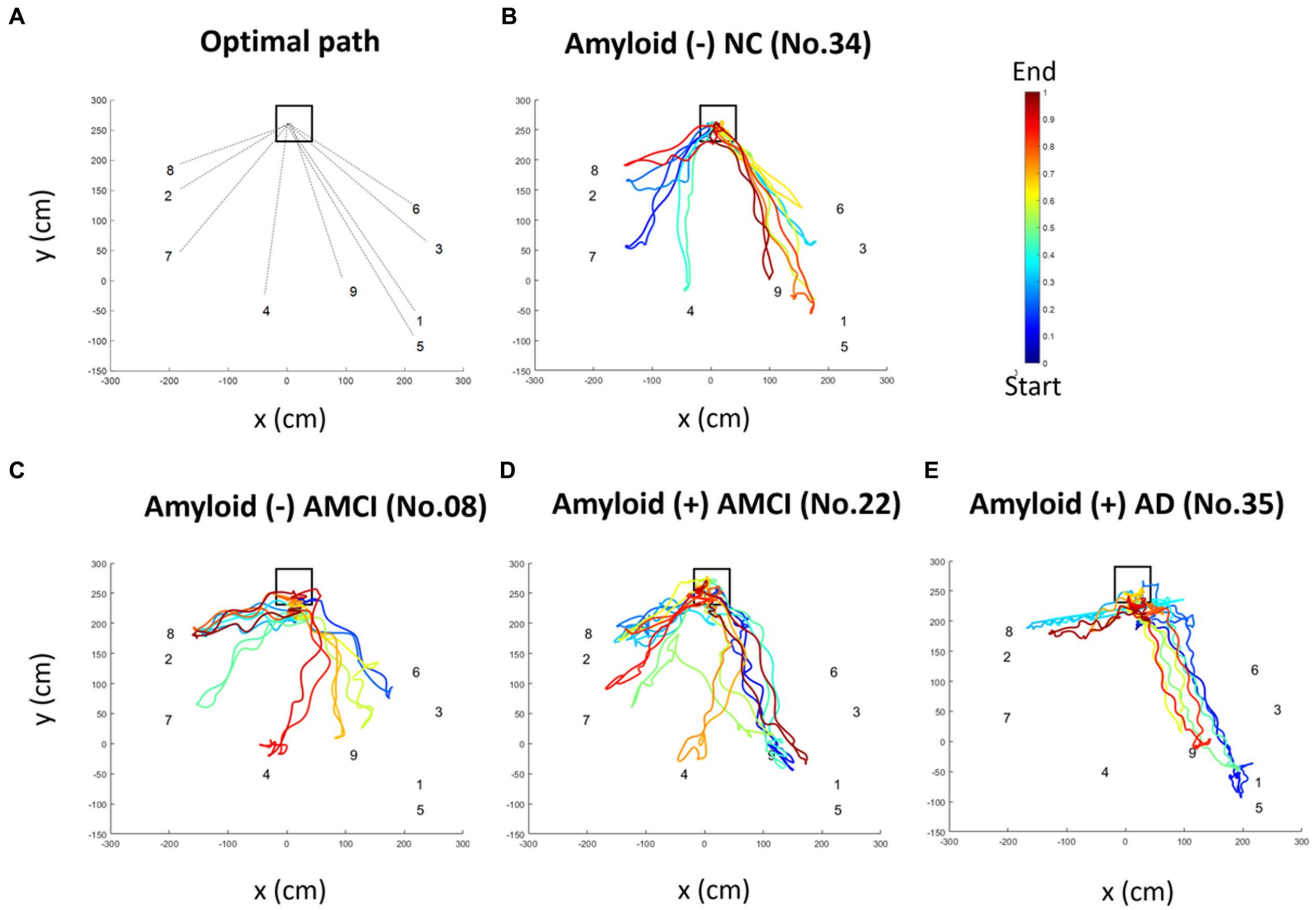
Representative pseudo-colored walking trajectory of each group. The square indicates the starting point, and the numbers indicate hidden places. The movement paths are colored according to the order of time. The return position was not identical to the location of the object because the participants returned after grasping the objects with the stroller in their hands. **(A)** The optimal movement path for each hidden location is indicated by dotted lines. Pseudo-colored movement paths of **(B)** amyloid-negative NC individuals, **(C)** amyloid-negative aMCI patients, **(D)** amyloid-positive aMCI patients, and **(E)** amyloid-positive AD patients. AD, Alzheimer’s disease; aMCI, amnestic mild cognitive impairment; NC, normal control.

Of the 46 validation group participants, trajectories were not recorded for four. We further excluded eight participants (four with AD, two with aMCI, and two NC) because their trajectory data were incomplete. Therefore, we estimated basic trajectory statistics, such as total distance, duration, and speed, for the remaining 34 participants (13 AD, 10 aMCI, and 11 NC) (Table 5). The total distance of the walking trajectory was not significantly different among three groups. The total duration of the walking trajectory was significantly different among three groups, but *post hoc* test showed that statistical significance existed only for AD vs. NC (*p* = 0.008). The mean speed of the walking trajectory was significantly higher in aMCI than in the AD (*p* = 0.035) and NC (*p* = 0.035) participants. A representative walking trajectory showed that patients with AD and aMCI wandered rather than going straight toward the hidden objects (Figure 4). To quantify the wandering pattern, we applied *trajectory data mining* such as outlier detection and stay point detection.

**Table 5:** Comparison of walking trajectory features among the three groups.

	Mean ± SD			*p* value	*p* value *(Post hoc)*			AD (*N* = 13)	aMCI (*N* = 10)	NC (*N* = 11)	AD vs. aMCI	AD vs. NC	aMCI vs. NC
*Basic features of movement path*							
Total distance (m)	65.20 **±** 17.19	69.07 **±** 11.08	57.94 **±** 6.59	0.121	0.999	0.777	0.105
Total duration (s)	369.09 ± 132.83	337.64 ± 87.32	246.74 ± 51.46	*0.013*	0.365	*0.008*	0.546
Mean speed (m/s)	0.16 ± 0.03	0.19 ± 0.04	0.16 ± 0.04	*0.03*	*0.035*	0.599	*0.035*
*Trajectory pattern mining*							
Number of outliers	3457.92 ± 1223.50	3191.30 ± 863.99	2318.09 ± 496.53	*0.026*	0.999	*0.015*	0.083
Distance of outliers (m)	0.52 ± 0.10	0.46 ± 0.06	0.40 ± 0.07	*0.012*	0.568	*0.002*	0.568
Number of stay points	30.62 ± 14.58	26.40 ± 6.65	13.73 ± 6.25	*0.002*	0.999	*0.003*	*0.019*

AD, Alzheimer’s disease; aMCI, amnestic mild cognitive impairment; NC, normal control. The italic values show significant differences at *p* values < 0.05.

**Figure 5:**
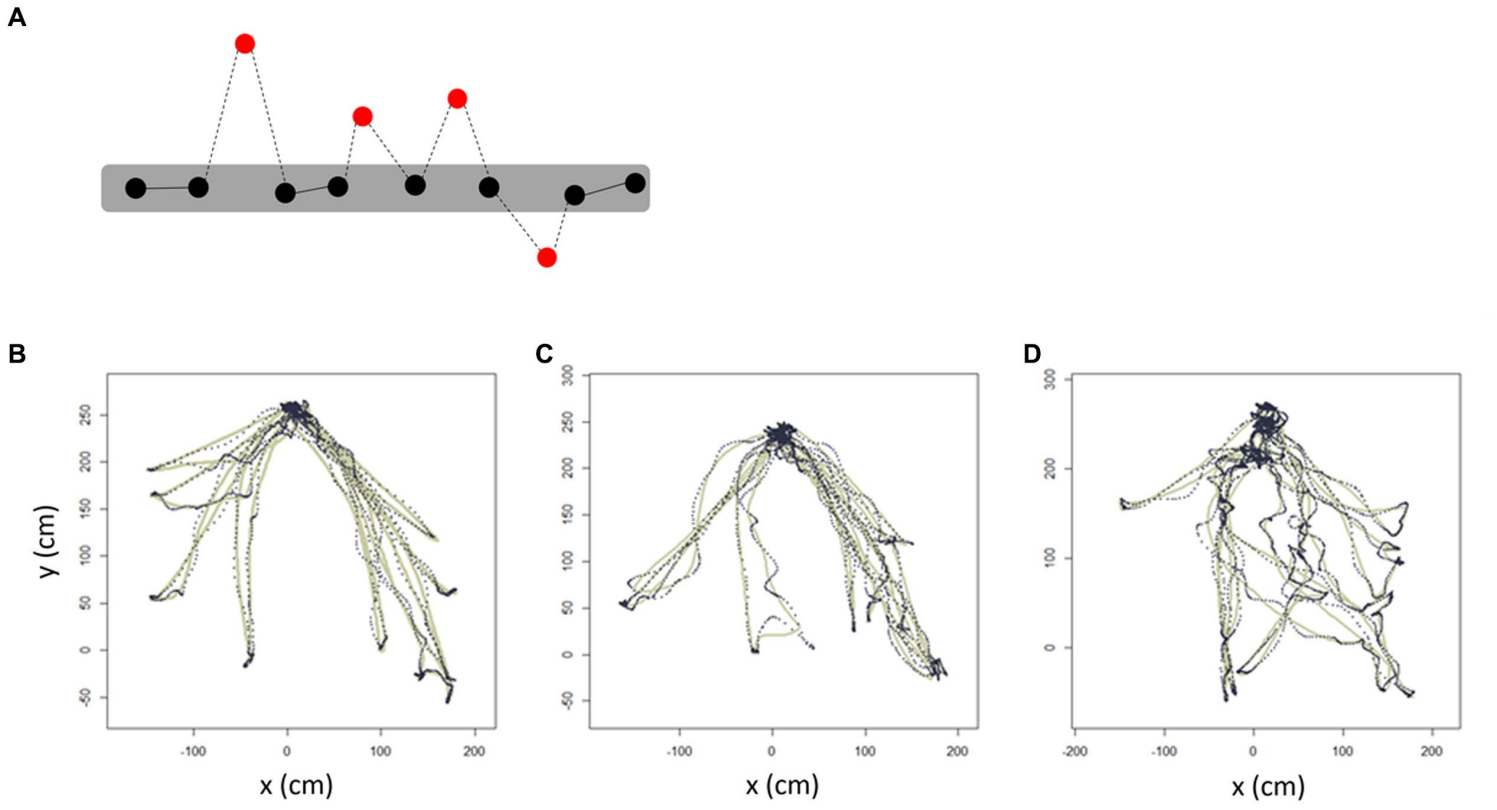
Trajectory outlier detection. Schematic diagram showing the trajectory-outlier detection method. **(A)** Red dots indicate *outliers* which are noise points in the estimated trajectory. Dotted lines indicate *the distance of outliers.* Representative trajectories of **(B)** NC, **(C)** aMCI, and **(D)** AD. The solid line represents the 95% CI for the parameter estimate using the Hessian matrix, resulting in Gaussian residuals, whereas the dotted line represents the individual walking trajectory. AD, Alzheimer’s disease; aMCI, amnestic mild cognitive impairment; NC, normal control.

Trajectory outliers were defined as points that were significantly different from the others in terms of a 95% CI for the parameter estimate using the Hessian matrix, resulting in Gaussian residuals. Representative diagrams of the outlier detection results in the AD, aMCI, and NC groups are shown in Figures 5A–D. We found that *the number of outliers* tended to be higher in AD and aMCI patients than in NC, but the statistical difference was only significant between the AD and NC (*p* = 0.015) groups. *The number of outliers* between the AD and aMCI groups were not significantly different. Similarly, *the distance of outliers* was significantly longer in AD than in NC (*p* = 0.002), but was not significantly different between the AD and aMCI groups (Table 5).

**Figure 6:**
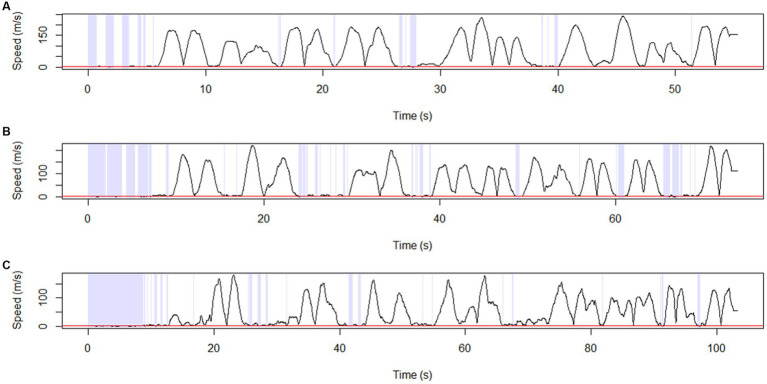
Stay point detection. Representative diagram showing stay-point detection results for **(A)** NC, **(B)** aMCI, and **(C)** AD. The points at which the solid black line (transformed trajectory) is adjacent to the red line represent the stay points. S*tay points* were defined as points where a participant remained stationary for 2 s within a distance of 0.3 meters. AD, Alzheimer’s disease; aMCI, amnestic mild cognitive impairment; NC, normal control.

Trajectory *stay points* were defined as points where a participant remained stationary for 2 s within a distance of 0.3 meters. Representative diagrams of the *stay-point detection* results in the AD, aMCI, and NC groups are shown in Figures 6A–C. We found that *the number of stay points* was significantly higher in AD and aMCI patients compared to NC (AD vs. NC: *p* = 0.003, aMCI vs. NC: *p* = 0.019) (Table 5).

The basic and trajectory pattern mining features of the movement path were not significantly different between the amyloid-negative and amyloid-positive aMCI groups (Supplementary Table S4).

## 4 Discussion

The HOT was created to reflect the symptoms of losing or misplacing objects and being unable to find them, which is one of the most frequent behaviors that patients with Alzheimer’s disease display in everyday life. We used a freely movable virtual environment and allowed participants to move to the hidden places. To evaluate various aspects of memory, the HOT consists of five subtests: *prospective memory, item free-recall*, *place free-recall*, *item recognition*, *and place-item matching*. In addition, our HOT implemented a photorealistic pleasant and comfortable living room to reflect real-life situations by misplacing objects at home using the benefits of VR.

We investigated visuospatial memory impairment in participants with AD, aMCI, and NC by analyzing HOT scores. Performance differed across all groups (AD, aMCI, and NC) on total HOT score and the *item recognition* subtest (Figures 3A,D). Both diagnostic groups (AD and aMCI) had lower scores than the NC group on measures of *item free-recall*, *place free-recall*, and *place-item matching tests* (Figures 3B,C,E). The *prospective memory test* only revealed a difference between the AD and NC groups (Figure 3F). As anticipated, HOT scores generally correlated with conventional neuropsychological measures of verbal and visuospatial memory and a few measures of executive function. Compared to the SVLT and RCFT delayed recall tests (Figures 3G,H), the HOT subtests, especially the item-place matching test, showed potential for better distinguishing AD and MCI groups from NC group. The *item free-recall* and *place free-recall tests* showed a significant degree of overlap among these measures, whereas the *place-item matching test* could be a good candidate for assessing target key features distinguishing early AD from normal aging. The *place-item matching test* tested associative memory by asking participants to recall the place where a particular object was hidden. Previous studies have found that associative memory, the ability to encode and recall relationships between unrelated items, is sensitive in the early stages of AD (Parra et al., 2010; Rentz et al., 2013; Papp et al., 2015). The *prospective memory test* (Figure 3F) was insufficient to differentiate between the groups. HOT scores did not show a significant correlation with age or education. The lack of age and education-related effects are not interpreted due to the small and heterogenous (AD, aMCI, and NC) sample.

Notably, we found subtle differences in the basic features of the walking trajectories among the groups. The total duration suggested that patients with AD wandered rather than moving straight toward the hidden objects. Based on the wandering pattern in patients with AD and aMCI, we performed trajectory pattern mining using *trajectory outlier* and *stay point detection* algorithms to quantify wandering of patients with AD and aMCI. Previous studies have investigated the trajectories of vehicles and people using trajectory pattern mining (Zheng, 2015). We applied these concepts to participants with AD and aMCI compared to NC, a group who did not exhibit impairment on neuropsychological tests. In terms of *trajectory outlier detection*, the greater *number and distance of outliers* in participants with diagnosed AD and aMCI indicated that participants with AD/aMCI frequently moved out of the estimated walking trajectory compared to NC participants. In terms of *stay point detection*, a greater number of *stay points* in the AD and aMCI groups indicated more frequently stopping or longer duration of staying while searching for hidden objects. A recent study applied *stay point detection* to demonstrate the behavioral phenotypes of patients with schizophrenia (Jongs et al., 2020). In our study, we applied these trajectory data-mining methods to the movement paths and found that outliers and stay points may represent wandering and hesitation instead of going straight to hidden objects in participants with AD and aMCI compared to NC.

Our HOT results did not distinguish between amyloid-negative and amyloid-positive aMCI subgroups. In terms of the total HOT score, amyloid-negative aMCI patients tended to have a higher score than amyloid-positive aMCI patients, but the difference did not reach statistical significance (Supplementary Table S3). In terms of the walking trajectory data (Figure 4), amyloid-positive aMCI patients appeared to have less direct trajectories towards the targets compared to amyloid-negative aMCI patients; however, the difference did not reach statistical significance (Supplementary Table S4).

There are several limitations. First, our results are preliminary and interpretations are constrained due to relatively small sample sizes. Therefore, future standardization studies with larger numbers of participants are warranted. Second, feasibility was not assessed using an observational checklist. While no participants discontinued the VR test due to cybersickness, and most participants, including dementia patients, provided positive feedback, a questionnaire rating for feasibility assessment will be necessary in the follow-up study. Despite its limitations, our study demonstrated the application of VR in exploring the cognition and other behaviors of individuals with AD and aMCI compared to controls. While our study primarily focused on using VR to detect visuospatial memory impairment commonly observed in patients with AD, there is potential to extend its use as an assessment tool for other cognitive domains such as learning and memory, perceptual-motor function, and executive function (Chua et al., 2019).

In conclusion, we developed a visuospatial memory test using VR based on patient complaints of losing or misplacing objects. Our preliminary results showed that the total HOT scores differed between the AD, aMCI, and NC groups. HOT scores were correlated with conventional neuropsychological test scores, specifically tests of memory and executive function, supporting the construct validity of this new measure’s ability to assess aspects of memory. This study suggests that the HOT may be a useful new measure of memory with a potentially less biased assessment approach and greater ecologically validity and with potential to incorporate more useful performance metrics for the assessment of visuospatial memory and other aspects of cognitive function in daily life.

## Data availability statement

The raw data supporting the conclusions of this article will be made available by the authors, without undue reservation.

## Ethics statement

The studies involving humans were approved by the institutional review board of the Samsung Medical Center. The studies were conducted in accordance with the local legislation and institutional requirements. Written informed consent for participation in this study was provided by the participants’ legal guardians/next of kin.

## Author contributions

DN, JHC, JC, and BL conceived and designed the study. KK performed the statistical analyses and wrote the first draft of the manuscript. JDC organized the database and wrote the sections of the manuscript. All authors contributed to the article and approved the submitted version.

## Glossary

**Table:** 

AD	Alzheimer’s disease
aMCI	amnestic Mild Cognitive Impairment
CDR	Clinical Dementia Rating
COWAT	Controlled Oral Word Association Test
FDR	False Discovery Rate
HMD	Head Mounted Display
HOT	Hidden Objects Test
K-BNT	Korean version of the Boston naming test
MMSE	Mini-Mental State Examination
MRI	Magnetic Resonance Imaging
NC	Normal Control
PET	Positron Emission Tomography
RCFT	Rey-Osterrieth Complex Figure Test
SNSB	Seoul Neuropsychological Screening Battery
SVLT	Seoul Verbal Learning Test
VR	Virtual Reality
