# Supplementary material for: Development and preliminary validation of a virtual reality memory test for assessing visuospatial memory
Source: Front Aging Neurosci. 2023 Nov 23;15:1236084. doi: 10.3389/fnagi.2023.1236084 (PMC10701378; doi:10.3389/fnagi.2023.1236084)
Supplement: Supplementary file 1 [file Table_1.DOCX]

**Development and Preliminary Validation of a Virtual Reality Memory Test for Assessing Visuospatial Memory**

**Ko Woon Kim, MD, PhD,^1,2^ Jong Doo Choi, MS, ^3^ Juhee Chin, PhD, ^4^ Byung Hwa Lee, PhD , ^4^ Jee Hyun Choi, PhD,^5^ Duk L. Na, MD, PhD ^4^***

*** Correspondence:** Duk L. Na, M.D., Ph.D. (e-mail: dukna@naver.com)

# Supplementary Tables

**Supplementary Table 1. The categorized top 223 words from frequently used Korean vocabulary**

| **Category (number)** | **The top 223 words from frequently used Korean vocabulary** |
| --- | --- |
| Tools and electronics (41) | mirror, ball, kite, doll, chess, guitar, cards, robot, pencil, fountain pen, ballpoint pen, chalk, ink, brush, eraser, knife, glue, tape, drawing, paint, colored paper, paper, pistol, gun, arrow, radio, microphone, telephone, receiver, camera, lamp, switch, lantern, stick, cane, club, hammer, saw, basket, bowl, computer |
| Foods (64) | apple, persimmon, tangerine, strawberry, pear, peach, watermelon, melon, grape, egg, galbi (ribs), garlic, sweet potato, root, ginseng, corn, bean, bean sprout, green onion, red bean, pumpkin, rice, sesame seeds, mushroom, barley, ramen, cold noodles, noodles, lunchbox, seaweed, kimbap, kimchi, kimchi stew, rice cake, flour, water, makgeolli (rice wine), beer, cola, milk, coffee, alcohol, bulgogi, bibimbap, bread, cake, songpyeon (rice cake), candy, ice, honey, seasoning, soybean paste, soy sauce, oil, sugar, salt, oyster, sashimi, fish, shrimp, squid, food, vegetable |
| Wearable things (22) | necklace, ring, shoes, sneakers, boots, coat, traditional Korean clothing (hanbok), pants, rubber band, bag, belt, gloves, jeans, wallet, perfume, vest, socks, glasses, lens, suit, handkerchief, cloth |
| Other things (96) | towel, mop, book, notebook, picture book, notepad, magazine, Dish, tray, chopsticks, kettle, lid, cup, bottle, wine glass, glass, spoon, button, silk, thread, needle, money, check, bankbook, dollar, coin, grindstone, cigarette, match, candle, charcoal, stone, pebble, umbrella, parasol, bag, envelope, stamp, letter, plastic, mail, soap, detergent, toothbrush, toothpaste, pottery, hair, flag, lock, seed, tissue, balloon, ticket, barrel, faucet, horn, video, star, pillow, wheel, mask, fan, rope, net, treasure, medal, droplet, bell, monster, bus, box, blanket, car, oxygen, gold, monument, motorcycle, window, sedan, bicycle, machine, airplane, ski, automobile, train, building, trash can, vehicle, plane ticket, bus ticket, refrigerator, mountain, chair, garbage, grass, toy, iron |

**Supplementary Table 2.** **Clinical information and HOT scores of development group**

|  | **Mean±SD** | | | ***P value (Post hoc)*** | | |
| --- | --- | --- | --- | --- | --- | --- |
|  | **AD**  **(N = 7)** | **aMCI**  **(N = 6)** | **NC**  **(N = 10)** | **AD vs. aMCI** | **AD vs. NC** | **aMCI vs. NC** |
| **Clinical information** | | | | | | |
| Age | 72 ± 8 | 78 ± 6 | 74 ± 7 | 0.437 | >0.999 | 0.817 |
| Education | 10.4 ± 5.3 | 12.0 ± 7.3 | 10.6 ± 5.3 | >0.999 | >0.999 | >0.999 |
| MMSE | 19 ± 6 | 26 ± 2 | 28 ± 2 | 0.132 | *<0.001* | 0.581 |
| CDR | 1.1 ± 0.4 | 0.5 ± 0.0 | 0.0 ± 0.0 | 0.201 | *<0.001* | *0.045* |
| **HOT scores (total scores)** | | | | | | |
| Item Free-recall test (9) | 1 ± 1 | 4 ± 2 | 6 ± 2 | 0.309 | *<0.001* | 0.224 |
| Place Free-recall test (9) | 3 ± 2 | 5 ± 2 | 6 ± 2 | 0.167 | *0.005* | >0.999 |
| Item Recognition test (9) | 3 ± 2 | 7 ± 1 | 9 ± 1 | 0.125 | *<0.001* | 0.265 |
| Place-item matching (9) | 0 ± 1 | 1 ± 1 | 4 ± 3 | 0.578 | *0.007* | 0.407 |
| Prospective memory test (3) | 0 ± 0 | 1 ± 1 | 1 ± 1 | 0.533 | 0.423 | >0.999 |
| Total Score (39) | 7 ± 4 | 17 ± 6 | 25 ± 6 | 0.136 | *<0.001* | 0.316 |

# AD, Alzheimer’s disease; aMCI, amnestic mild cognitive impairment; CDR, Clinical Dementia Rating; HOT, Hidden Objects Test; NC, normal control; MMSE, Mini-Mental State Examination.

**Supplementary Table 3. Comparison of HOT scores between amyloid negative and positive aMCI.**

|  | **Mean±SD** | | ***P*** |
| --- | --- | --- | --- |
|  | **aMCI (-)**  **(N = 2)** | **aMCI (+)**  **(N = 7)** | **aMCI (-) vs. aMCI (+)** |
| Item Free-recall test (9) | 6 ± 1 | 3 ± 1 | 0.111 |
| Place Free-recall test (9) | 6 ± 1 | 5 ± 1 | 0.889 |
| Item Recognition test (9) | 8 ± 1 | 7 ± 1 | 0. 889 |
| Place-item matching (9) | 2 ± 1 | 1 ± 1 | 0.500 |
| Prospective memory test (3) | 2 ± 1 | 0 ± 0 | 0.056 |
| Total Score (39) | 23 ± 2 | 17 ± 3 | 0.111 |

aMCI (-), amyloid-negative amnestic mild cognitive impairment; aMCI (+), amyloid-positive amnestic mild cognitive impairment; HOT, hidden object test.

**Supplementary table 4. Comparison of walking trajectory features between amyloid negative and positive aMCI.**

|  | **Mean±SD** | | ***P*** |
| --- | --- | --- | --- |
|  | **aMCI (-)**  **(N = 2)** | **aMCI (+)**  **(N = 5)** | **aMCI (-) vs. aMCI (+)** |
| **Basic features of movement path** | | | |
| Total distance (m) | 6550.96 (763.08) | 6823.62 (1287.06) | 0.999 |
| Total duration (s) | 78.61 (5.42) | 93.34 (26.59) | 0.999 |
| Mean speed (m/s) | 0.83 (0.04) | 0.75 (0.10) | 0.381 |
| **Trajectory Pattern Mining** | | | |
| Number of outliers | 2754.00 (162.63) | 3291.20 (1056.46) | 0.999 |
| Distance of outliers (m) |  |  |  |
| Number of stay points | 27.00 (7.07) | 26.60 (7.16) | 0.999 |

aMCI (-), amyloid-negative amnestic mild cognitive impairment; aMCI (+), amyloid-positive amnestic mild cognitive impairment.
